# Structure Identification of Germplasm Resources of Lotus with High Resistant Starch

**DOI:** 10.3390/polym18141737

**Published:** 2026-07-15

**Authors:** Bin Wang, Zelin Li, Fenglin Zhu, Liangbo Yang, Xingwen Zheng, Shoulei Yan, Ying Diao, Zhongli Hu

**Affiliations:** 1 College of Life Science and Technology, Wuhan Polytechnic University, Wuhan 430023, China; wangx10050115@163.com (B.W.); lizelin0118@foxmail.com (Z.L.); 2 Honghu Lotus Root Industry Research Institute, Honghu 433200, China; zhufenglin428@163.com (F.Z.); yanshoulei1225@mail.hzau.edu.cn (S.Y.); 3 Guangchang Research School of White Lotus, Guangchan 344900, China; yangliangb@126.com (L.Y.); xwzheng@126.com (X.Z.); 4 College of Food Science and Technology, Huazhong Agricultural University, Wuhan 430023, China

**Keywords:** lotus, germplasm, resistant starch, structural characterization

## Abstract

This study focused on resistant starch (RS) from lotus, analyzing its content and structural characteristics across different lotus germplasms. Detection using the Dual-Wavelength Colorimetric Method revealed the following findings: The proportion of amylose in fresh lotus rhizomes was higher than that in fresh lotus seeds. The average content of resistant starch in fresh lotus rhizomes was also higher than that in fresh lotus seeds. After cooking, the resistant starch content of both lotus rhizomes and seeds decreased, with lotus seeds retaining a relatively higher amount of resistant starch post-cooking. Structural characterization showed that the surface of resistant starch from lotus seeds exhibited a grooved morphology, whereas that from lotus rhizomes appeared as irregular clusters. Additionally, the structure of raw resistant starch was more compact than that of cooked resistant starch. Fourier Transform Infrared (FTIR) Spectroscopy and X-ray diffraction (XRD) analyses confirmed that all lotus-derived resistant starches formed a type-C crystalline structure, with the crystallinity of resistant starch from lotus seeds being higher than that from lotus rhizomes. This study provides a theoretical foundation for breeding of high-RS lotus varieties and for the effective utilization of lotus germplasm. This work systematically compares RS composition and multi-scale structural differences between two edible lotus organs across a large germplasm population, clarifying the structural basis of RS variation, which distinguishes this study from previous single-material lotus RS research.

## 1. Introduction

Lotus (*Nelumbo nucifera*) is a significant aquatic economic crop recognized for its ornamental, edible, and medicinal properties [1,2]. Systematic exploration and efficient utilization of its germplasm are essential for advancing scientific research and supporting the industrial economy [3]. The primary edible components of the lotus are its seeds (lotus seeds) and rhizomes (lotus rhizome), both of which are rich in starch. Starch content is a critical factor influencing their nutritional quality and processing performance, directly affecting the taste, texture, and storage stability of lotus seeds and roots, as well as their suitability and functional performance in food processing [4]. Notable genotypic variation in starch content exists among different lotus germplasm. These variations not only impact the quality of final products and the selection of processing technologies but may also be associated with the plants’ growth and development traits, ecological adaptability, and stress resistance. For instance, lotus seeds with high starch content provide substantial carbon sources and energy reserves for seed germination and early seedling growth, while the starch content in lotus rhizome influences their cooking characteristics, powdery texture, and the yield and quality of processed products such as lotus root starch and lotus root slices [5].

With the increasing popularity of modern nutrition and healthy dietary concepts, lotus starch—a multifunctional carbohydrate derived from natural plants—has attracted growing attention for its nutritional properties and physiological functions [6]. In particular, RS, a starch fraction that resists digestion and absorption in the small intestine, exhibits physiological effects akin to dietary fiber, such as regulating intestinal flora, delaying postprandial blood glucose spikes, and enhancing satiety, thereby providing multiple health benefits [7,8]. However, systematic evaluations of starch components—especially amylose, amylopectin, and RS—in lotus germplasm remain limited. Furthermore, there is a lack of in-depth analysis regarding the variation patterns of starch content and its structural basis across different germplasms. Therefore, conducting comprehensive research on starch content in lotus germplasm and identifying superior varieties with high starch content or specific component characteristics (e.g., elevated RS levels) holds significant theoretical and practical value for advancing genetic breeding, variety improvement, and the efficient utilization of the entire lotus industrial chain.

Based on this premise, the study utilized diverse lotus germplasm as materials, collecting samples of lotus seeds and roots. The contents of amylose and amylopectin were determined using the Dual-Wavelength Method, and in vitro simulated digestion analysis of RS was conducted in accordance with relevant group standards [9,10,11]. The aim was to comprehensively evaluate the starch composition characteristics across different germplasms, thereby identifying superior lotus germplasms with high RS content and providing a material basis for subsequent functional variety breeding and industrial development.

In recent years, research on lotus RS has made significant progress in content determination and physiological function evaluation. Multiple studies have confirmed that lotus RS exhibits beneficial biological activities, including the regulation of blood glucose and lipid levels, and the improvement in intestinal health [12]. Although existing literature has systematically summarized the classification system, basic properties, and common preparation methods of RS, the intrinsic relationship between its content levels and structural attributes remains unclear [13]. This gap limits the precise breeding of high-RS lotus varieties and the targeted development of related products. Additionally, the structural characteristics, modification methods of RS, and its potential applications in food systems have become prominent research hotspots.

Some scholars have adopted a strategy of combining multiple analytical techniques to systematically elucidate the “structure-function” relationship of RS. However, research on the structural analysis of lotus RS remains relatively limited. To clarify the intrinsic connection between the content and structure of lotus RS, this study selected representative germplasms with high RS content and employed multi-scale structural characterization techniques, including Scanning electron microscopy (SEM), Fourier Transform Infrared Spectroscopy (FTIR), and X-ray diffraction (XRD). These methods were used to systematically compare and analyze differences in particle morphology, molecular conformation, and crystal structure between natural starch and processed RS in lotus seeds and roots. Through the comprehensive application of these techniques, the structural differences in RS among various lotus germplasm, as well as the mechanisms by which processing influences the structural evolution of RS, can be revealed at multiple levels—from microscopic to macroscopic—thereby establishing a structural foundation for understanding the physicochemical properties and physiological functions of lotus RS.

## 2. Materials and Methods

### 2.1. Plant Material

In this study, a total of 155 lotus germplasm were selected, comprising 95 rhizome lotus accessions and 60 seed lotus accessions, all collected from the Lotus Research Base of Wuhan University in Ezhou, Hubei Province (See Supplementary Tables S1 and S2 for full germplasm metadata, including germplasm code, genetic background, cultivation environment and sampling year. Germplasm codes starting with A represent rhizome lotus, while codes starting with B represent seed lotus.). The lotus seeds utilized were naturally sun-dried to constant weight under ambient conditions. For the determination of amylose, amylopectin, and RS contents, healthy and intact second internodes of the main rhizome, as well as mature dried lotus seeds without insect damage or mildew, were selected as test samples. All samples were subjected to three biological replicates for subsequent index determination and structural characterization.

### 2.2. Determination of Moisture Content

The direct drying method specified in GB/T 5009.3-2010 was adopted [14]: the sample powder (passed through an 80-mesh standard sieve) was weighed and recorded as mass a (g), then placed in a forced-air drying oven (Shanghai Bluepard Instruments Co., Ltd. Shanghai, China) at 105 °C to dry until constant weight, and the constant mass was recorded as b (g). The moisture content was calculated according to the following formula.

(1) $$\mathrm{moisture}\left(\%\right)=\frac{a-b}{a}\times 100\%$$

### 2.3. Determination of Amylose and Amylopectin Contents

Amylose–Iodine and amylopectin–iodine Complex Dual-Wavelength Colorimetric Determination Method (specially optimized for high-starch lotus samples) [10].

(2) $$\mathrm{Amylose}\left(\%\right)=C_{A}\times 50\times \frac{100}{5\times 100}\times (1-W_{1}-W_{2})\times 100\%$$

(3) $$\mathrm{Amylopectin}\;(\%)=C_{Ap}\times 50\times \frac{100}{\;5\times 100}\times (1-W_{1}-W_{2})\times 100\%$$

In the formula, C_A_ = amylose concentration (mg/mL); C_Ap_ = amylopectin concentration (mg/mL); W_1_ represents the moisture content (%); W_2_ represents the total crude fat content (%).

### 2.4. Determination of Resistant Starch Content

Reference: content indexes and detection methods for high-resistant-starch rice used in cooking and processing (T/CI 037-2022) [11].

#### 2.4.1. Determination of Retrograded Cooked RS

Accurately weigh 100 mg sieved starch powder and transfer it to the bottom of a 50 mL round-bottom centrifuge tube. An appropriate volume of distilled water was added to fully moisten the powder, followed by cooking in an electric rice cooker (Joyoung Co., Ltd., Jinan, China) for 20 min and heat preservation for another 10 min. After cooling to ambient temperature, 2 mL of 0.1 mol/L KCl-HC (Sinopharm Chemical Reagent Co., Ltd., Shanghai, China) l buffer (pH 1.5) was added, and the mixture was fully homogenized with a tissue homogenizer. Subsequently, 100 μL of 10% (*w*/*v*) pepsin solution (Shanghai Macklin Biochemical Co., Ltd., Shanghai, China) was supplemented, and the sealed centrifuge tube was incubated in a thermostatted shaking incubator at 37 °C with shaking speed of 40 rpm for 1 h to simulate gastric digestion.

After gastric digestion, 4 mL of 0.1 mol/L sodium maleate buffer (Shanghai Yuanye Bio-Technology Co., Ltd., Shanghai, China) (pH 6.0) was added and mixed thoroughly, followed by 4 mL of composite digestive enzyme working solution containing 10 mg/mL pancreatic α-amylase (Shanghai Yuanye Bio-Technology Co., Ltd., Shanghai, China) and amyloglucosidase (Shanghai Macklin Biochemical Co., Ltd., Shanghai, China) with enzymatic activity of 3 U/mL. The tube was tightly capped and incubated in a thermostatted shaker (Tianjin Labotery Laboratory Apparatus Co., Ltd., Tianjin, China) at 37 °C with continuous shaking at 30 rpm for 16 h to simulate small intestinal digestion.

Upon completion of digestion, the mixture was centrifuged at 1500× *g* for 10 min, and the supernatant was discarded. A total of 2 mL of 50% (*v*/*v*) ethanol (Sinopharm Chemical Reagent Co., Ltd., Shanghai, China) was added to resuspend the precipitate via vortex (IKA, Guangzhou, China) oscillation, followed by gentle mixing with additional 6 mL of 50% (*v*/*v*) ethanol. The mixture was centrifuged at 4000 rpm for 10 min. The precipitate was washed twice repeatedly using 50% (*v*/*v*) ethanol, and the supernatant was discarded after each centrifugation step. The centrifuge tube was inverted to drain residual solvent, and filter paper was used to gently wipe off excess liquid attached to the inner tube wall.

The precipitate was fully resuspended in 1 mL distilled water, mixed with 1 mL of 4 mol/L KOH (Sinopharm Chemical Reagent Co., Ltd., Shanghai, China) solution, and oscillated in an ice-water bath for 20 min to solubilize RS granules. After ice bath treatment, 8 mL of 1.2 mol/L sodium acetate (Sinopharm Chemical Reagent Co., Ltd., Shanghai, China) buffer was added and blended evenly. Immediately afterward, 0.1 mL amyloglucosidase solution with total activity of 3300 U was supplemented, mixed uniformly, and incubated in a 50 °C water bath for 30 min; the tube was taken out and shaken thoroughly every 10 min during incubation.

After incubation, the outer wall of the centrifuge tube was wiped dry, and the mixture was centrifuged at 4000 rpm for 10 min. A volume of 10 μL supernatant was transferred into a 96-well microplate, mixed with 190 μL GOPOD (Shanghai Yuanye Bio-Technology Co., Ltd., Shanghai, China) colorimetric reagent, and incubated at 50 °C for 20 min before cooling to room temperature. The absorbance was measured at 510 nm using a microplate reader, with blank control samples set for baseline correction.

#### 2.4.2. Determination of Native Raw RS

Accurately weigh 100 mg sieved starch powder and transfer it to a 50 mL round-bottom centrifuge tube. No thermal cooking pretreatment was carried out for native raw RS samples. Directly add 2 mL of 0.1 mol/L KCl-HCl buffer (pH 1.5) and perform homogenization. All subsequent digestion, washing, solubilization, enzyme hydrolysis and colorimetric detection procedures were identical to those described for retrograded cooked RS in Section 2.4.1.

### 2.5. Preparation of Natural Starches from Lotus Seeds and Lotus Rhizomes

To begin, take 2 kg of fresh lotus seeds and lotus rhizomes. For the lotus seeds, remove the testa and cores; for the lotus rhizomes, peel and cut them into pieces [15]. Next, place the processed lotus seeds and rhizomes into a juicer (Midea Group Co., Ltd., Hangzhou, China) along with twice their weight in water for crushing. Pass the crushed mixture through a sieve and allow the resulting solution to stand for 5 h. After this period, decant the supernatant and wash the sediment. Allow the sediment to stand for an additional 5 h, then decant the supernatant once more. Wash the surface of the sediment and place it in an oven at 50 °C to dry thoroughly. Consequently, the natural starches from the lotus seeds and rhizomes are obtained.

### 2.6. Preparation of Resistant Starch from Lotus Seeds and Lotus Rhizomes [16]

To prepare the crude cooked RS, begin by placing 5 g of natural starch into a centrifuge tube and mixing it with an appropriate volume of distilled water to create starch milk. Subsequently, transfer the starch milk into a high-pressure autoclave (Shanghai Sanshen Medical Appliance Co., Ltd., Shanghai, China) and subject it to heat treatment at 111 °C for 10 min to facilitate starch gelatinization [17]. Once gelatinization is complete, remove the mixture and allow it to cool to room temperature before refrigerating it at 4 °C for 6 h. After this period, extract the retrograded starch and place it in an oven set to 50 °C for drying. Following drying, crush and sieve the starch to obtain the crude cooked RS. For the preparation of crude raw RS, simulate human digestion through an in vitro experiment. Begin by subjecting the raw starch to overnight digestion with salivary amylase, pepsin, and pancreatic amylase, during which non-RS components are hydrolyzed into glucose. Finally, use ethanol to terminate the reaction and dry the resulting residue to obtain the crude raw RS.

### 2.7. Scanning Electron Microscopy

Uniformly spread RS powder samples on conductive double-sided adhesive tape, followed by gold-sputtering coating to form a 10 nm conductive gold film layer. Two accelerating voltages were set for hierarchical observation: low voltage 3.0 kV for granule surface fine-texture imaging at 10,000× magnification; 10–20 kV for overall granule distribution scanning [18,19].

Stable experimental detection parameters: electron beam current maintained at 95–115 μA, vacuum degree ≤ 8.0 × 10^−3^ Pa. All samples were observed in triplicate, and representative micrographs were selected for presentation.

### 2.8. Fourier Transform Infrared Spectroscopy

RS sample powder was mixed with potassium bromide (KBr) at a mass ratio of 1:100, fully ground in an agate mortar under infrared lamp irradiation to remove free water and crystal water interference on characteristic absorption peaks [20]. After homogeneous mixing, the powder was pressed into transparent thin slices under vacuum compression. Spectral acquisition was performed in transmission mode at room temperature, with resolution set to 4 cm^−1^ and scanning wavenumber range of 400–4000 cm^−1^. All raw spectra were subjected to baseline correction and standard normalization before peak fitting analysis, with peak assignments referenced to the published starch FTIR literature [21].

### 2.9. X-Ray Diffraction

XRD analysis of RS samples was conducted over a 2θ scanning range of 5° to 50°. Diffraction patterns were collected using Cu-Kα radiation source equipped with a nickel filter and scintillation detector. Instrument operating parameters: tube voltage 40 kV, tube current 30 mA, divergence slit width 0.25 mm, K-Alpha1 wavelength 1.78901 Å, K-Alpha2 wavelength 1.7929 Å, K-Alpha2/K-Alpha1 intensity ratio = 0.5, continuous scanning rate 0.02°/min [22].

Relative crystallinity was quantitatively calculated via peak area integration method using MDI Jade 6.5 software: Relative crystallinity (%) = (Area of crystalline diffraction peaks/Total diffraction area) × 100%. Three parallel scanning replicates were performed for each sample to reduce detection error.

## 3. Results and Discussion

### 3.1. Quality Evaluation of Lotus Germplasm

#### 3.1.1. Determination Results of Starch Quality in Lotus Rhizome Germplasm

The quality evaluation results of 95 lotus rhizome germplasm are presented Supplementary Table S3. The moisture content of the lotus rhizomes ranged from 51.71% to 87.84%, with germplasm B278 exhibiting the highest moisture content and germplasm A091 the lowest; the average moisture content was 67.62%. The raw RS content of the 95 lotus rhizome germplasm varied from 1.03% to 20.40%, with germplasm A345 having the highest content and germplasm B350 the lowest, resulting in an average of 13.86%. The cooked RS content ranged from 0.63% to 8.07%, with germplasm B189 showing the highest content and germplasm A458 the lowest, yielding an average of 1.89%. Regarding the amylose and amylopectin contents of the 95 lotus rhizome germplasm, the amylose content spanned from 10.83% to 36.99%, with germplasm A223 having the highest amylose content and germplasm B167 the lowest; the average amylose content was 23.02%. The amylopectin content ranged from 63.01% to 89.17%, where germplasm B167 had the highest amylopectin content and germplasm A223 the lowest, with an average of 76.98%.

#### 3.1.2. Determination Results of Starch Quality in Lotus Seed Germplasm

The quality evaluation results of 60 dried lotus seed germplasm are presented Supplementary Table S4. The moisture content of the dried lotus seeds ranged from 2.98% to 6.83%, with germplasm A336 exhibiting the highest moisture content and germplasm A045 the lowest; the average moisture content was 5.55%. The RS content among the 60 lotus seed germplasm varied significantly, with raw RS content ranging from 1.34% to 14.53%. Germplasm A328 had the highest RS content, while germplasm B200 had the lowest, resulting in an average of 6.15%. The cooked RS content ranged from 1.08% to 5.24%, with germplasm B481 showing the highest content and germplasm B065 the lowest, yielding an average of 2.20%. Regarding the amylose and amylopectin contents, the amylose content spanned from 7.66% to 12.06%, with germplasm B132 and B279 having the highest amylose content and germplasm B179 the lowest; the average amylose content was 9.38%. The amylopectin content ranged from 87.94% to 92.34%, where germplasm B179 had the highest amylopectin content, while germplasm B132 and B279 had the lowest, resulting in an average of 90.62%.

### 3.2. Analysis of Overall Differences in Resistant Starch Content Among Lotus Germplasm

The overall difference analysis was conducted on the RS contents presented in Table 1 and Table 2. As shown in Table 1 and Table 2, significant differences in RS content were observed between lotus rhizomes and lotus seeds. Among the 95 lotus rhizome germplasm, the raw RS content ranged from 1.03% to 20.40%, with a standard deviation of 3.93%. The cooked RS content varied from 0.63% to 8.07%, with a standard deviation of 1.33%. In the case of the 60 lotus seed germplasm, the raw RS content ranged from 1.34% to 14.53%, with a standard deviation of 3.91%, while the cooked RS content ranged from 1.08% to 5.24%, with a standard deviation of 0.91%. An analysis of variance (ANOVA) was performed on the RS content of the lotus germplasm. The results indicated no significant difference in RS content; however, a high coefficient of genetic variation was observed. The existing lotus germplasm demonstrate considerable genetic potential regarding RS content, and their rich genetic diversity offers substantial opportunities for breeding and selection efforts.

The ChiPlot (https://www.chiplot.online/, accessed on 15 July 2026) software was utilized to perform a correlation analysis of five quality indicators across 155 lotus germplasm. The Pearson correlation coefficient was employed to quantify the strength of the correlations, with values ranging from −1 to 1. A value closer to 0 indicates a weaker correlation, while values approaching −1 or 1 signify stronger correlations. The sign of the coefficient indicates the direction of the correlation: a positive sign denotes a positive correlation, represented in orange, whereas a negative sign indicates a negative correlation, represented in green.

**Figure 1:**
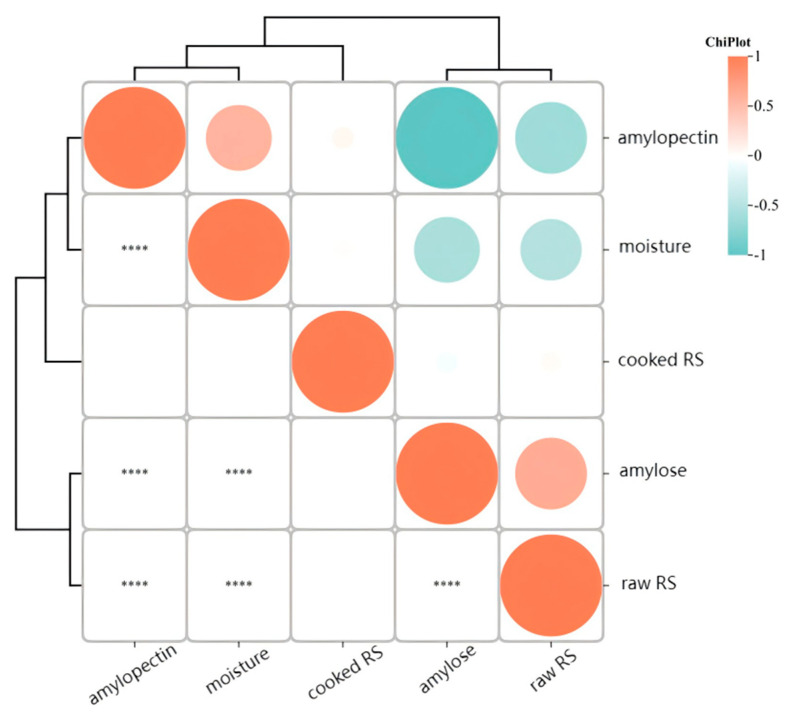
Correlation analysis of lotus root. Note: “****” indicates *p* < 0.00001. Orange represents a positive correlation and green represents a negative correlation.

As illustrated in Figure 1, for lotus rhizomes, moisture content exhibited a positive correlation with both amylopectin content and cooked RS content. This suggests that higher moisture content in lotus rhizomes is associated with increased amylopectin and cooked RS content. Furthermore, amylose content demonstrated a positive correlation with raw RS content, indicating that higher amylose content corresponds to higher raw RS content.

**Figure 2:**
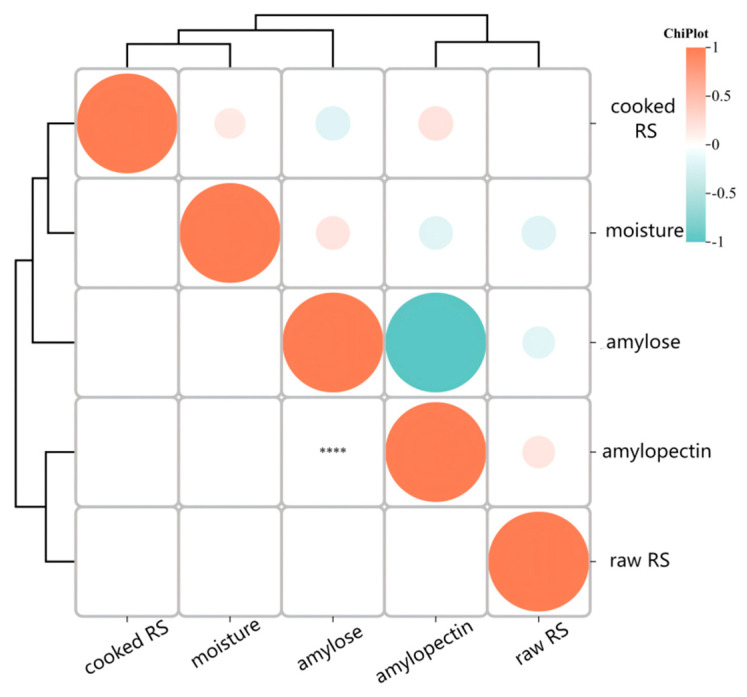
Correlation analysis of lotus seed. Note: “****” indicates *p* < 0.00001. Orange represents a positive correlation, and green represents a negative correlation.

As depicted in Figure 2, for lotus seeds, moisture content was positively correlated with both amylose content and cooked RS content, while amylopectin content showed a positive correlation with both raw RS content and cooked RS content.

### 3.3. Cluster Analysis

The ChiPlot software was utilized to conduct cluster analysis on 155 lotus germplasm. The upper section of the figure presents a dendrogram that illustrates the cluster structure, displaying the affinities among different samples through branches; the proximity of the branches indicates the similarity of the samples concerning the characteristics of the corresponding indicators. The lower section features a heatmap, with horizontal columns representing the five major indicators and vertical columns representing the samples. The heatmap employs a color gradient to convey the magnitude of values, as indicated by the color bar on the right, where values transition from cool tones to warm tones, thereby intuitively illustrating the numerical differences among each sample across various indicators.

The dendrogram reveals that the lotus germplasm are categorized into distinct clusters. Germplasm within the same cluster exhibit similar overall performance across multiple indicators, suggesting that they may share comparable genetic backgrounds or growth environments. For instance, samples that are closely clustered may belong to the same variety or be cultivated under similar ecological conditions.

**Figure 3:**
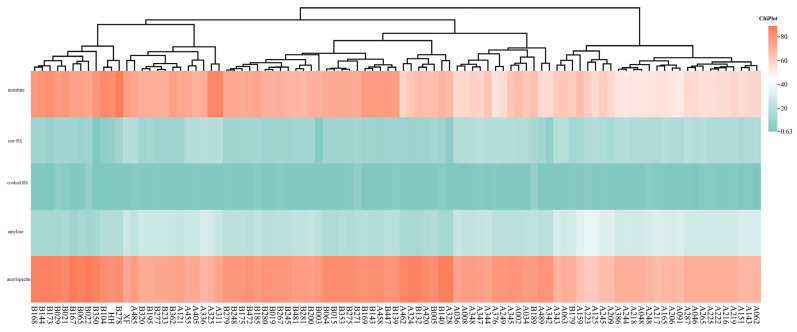
Cluster analysis of lotus root. Note: The left side represents Category I, and the right side represents Category II.

**Figure 4:**
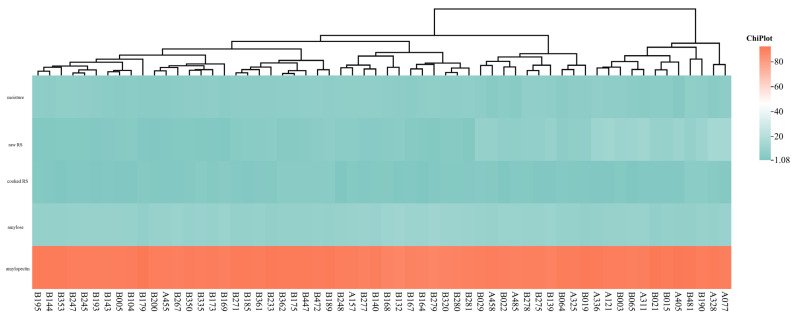
Cluster analysis of lotus seed. Note: The left side represents Category I, and the right side represents Category Scanning electron microscopy.

As illustrated in Figure 3, the 95 lotus rhizome germplasm are classified into two primary categories (Category I and Category II): Category I comprises 76 germplasm, which can be further subdivided into two subcategories; Category II includes 19 germplasm, which can also be divided into two subcategories. Similarly, Figure 4 demonstrates that the 60 lotus seed germplasm are categorized into two major groups (Category I and Category II): Category I contains 48 germplasm, which can be further divided into two subcategories, while Category II encompasses 12 germplasm.

**Figure 5:**
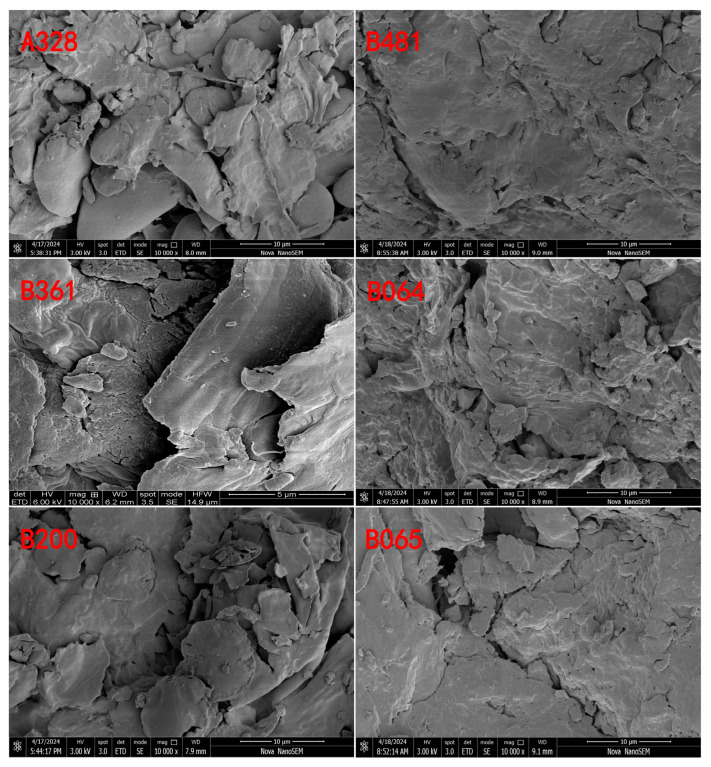
Scanning image of lotus seed Scanning electron microscopy (SEM). Note: The left column represents raw RS; the right column represents cooked RS. From top to bottom, the three samples are arranged in descending order of resistant starch content (i.e., high, medium, and low).

Scanning electron microscopy (SEM) provides valuable insights into the surface morphology and microstructure of RS granules derived from lotus germplasm. The morphological structure of lotus seed RS was examined at a magnification of 10,000×, with the results illustrated in Figure 5.

The SEM images reveal that the surface of lotus seed RS granules exhibits intricate details: rather than being completely smooth, the surface is rough, characterized by fine textures and folds, predominantly displaying groove-like or pore-like structures. These textures may be associated with the synthesis and crystallization processes of starch. Additionally, some granules display tiny holes on their surfaces, which may influence the interaction between starch and external substances, such as the binding of digestive enzymes.

Furthermore, SEM observations indicate a degree of aggregation among the starch granules, primarily resulting from mutual adhesion, leading to the formation of aggregates of varying sizes. A comparison of the raw and cooked lotus seed RS, presented in the left and right columns, clearly demonstrates that raw RS appears smoother and more distinct, while the boundaries between cooked RS granules become less defined following high-temperature treatment.

**Figure 6:**
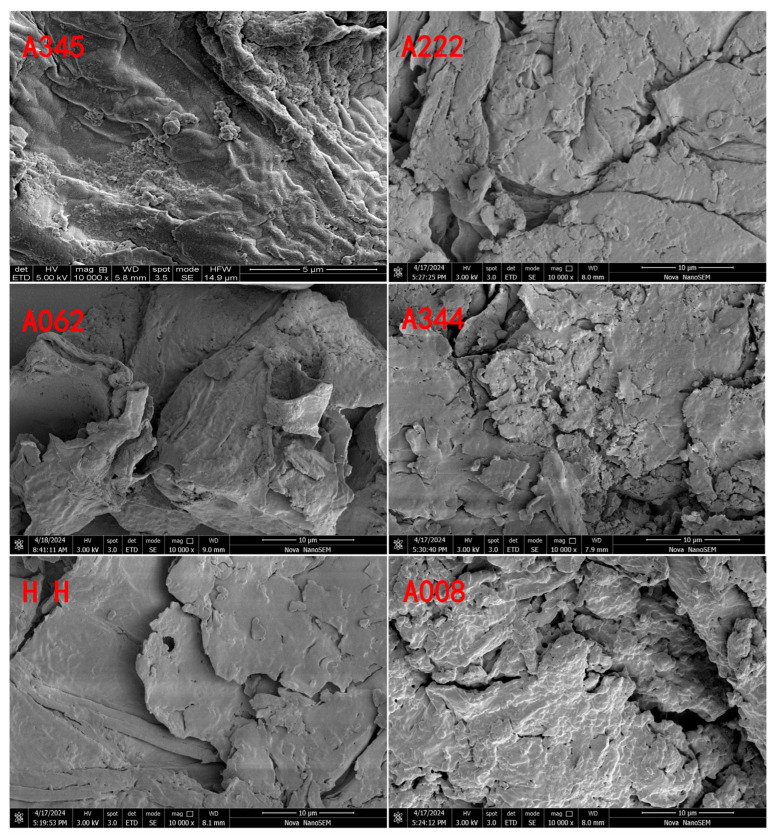
Scanning image of lotus root Scanning electron microscopy (SEM). Note: The left column represents raw RS; the right column represents cooked RS. From top to bottom, the three samples are arranged in descending order of resistant starch content (i.e., high, medium, and low).

The morphological structure of lotus root RS was examined at a magnification of 10,000×, with the results presented in Figure 6. Scanning electron microscope (SEM) images reveal irregular clustered structures, suggesting that the treatment may have damaged the starch granules and cleaved the glucan chains into shorter amylose fragments. These shorter amylose chains subsequently aggregated to form irregular clustered granules with a flaky surface. The observed results align with previously reported RS structures derived from lentil and pea starches. A comparison of raw and cooked RS from lotus root clearly demonstrates that the structure of raw RS is significantly more compact, whereas that of cooked RS is considerably looser.

### 3.4. Fourier Transform Infrared Spectrum

**Figure 7:**
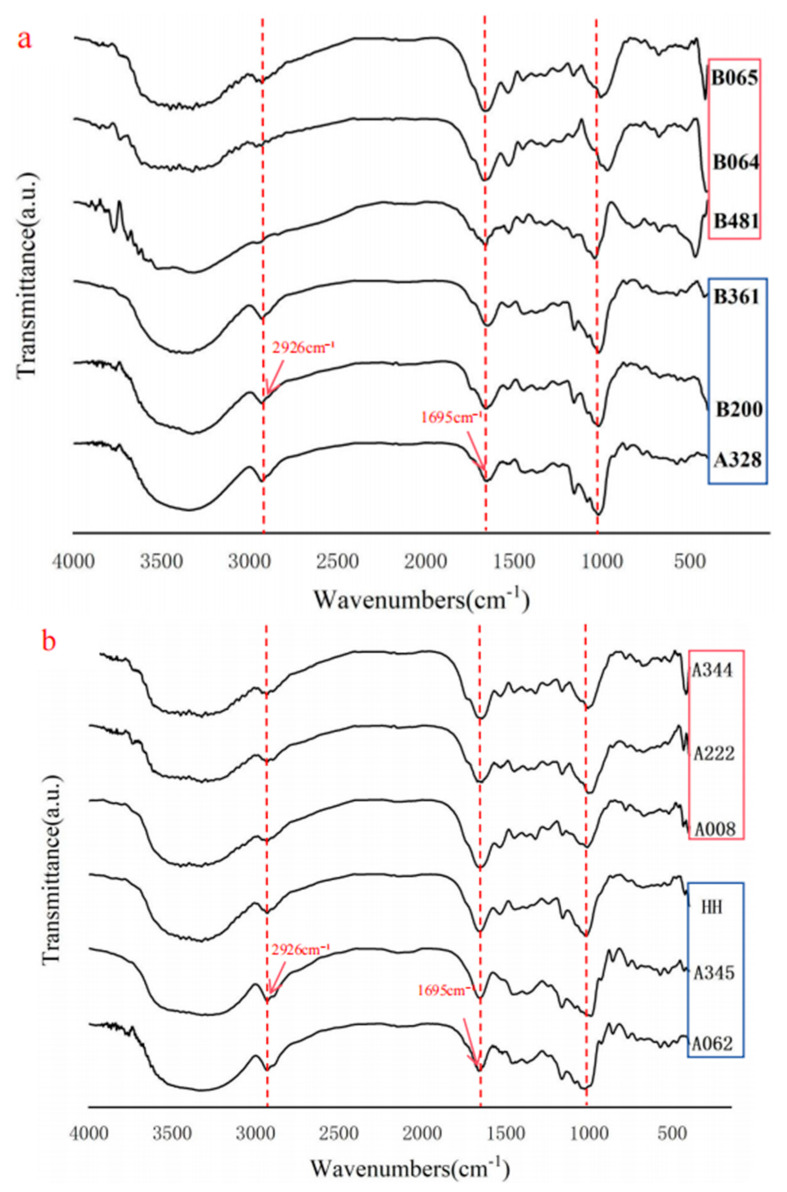
Infrared absorption spectrum. Note: (**a**) Lotus seeds; (**b**) lotus rhizome. The samples in the red boxes are cooked RS, and the samples in the blue boxes are raw RS. The red dashed line is the C−H stretching vibrations at 1000 cm^−1^, 2926 cm^−1^, and 1695 cm^−1^.

Fourier Transform Infrared (FTIR) Spectroscopy was employed to identify the chemical functional groups and bonding information of RS derived from lotus root. In the FTIR spectrum (Figure 7), RS exhibited strong C−H stretching vibrations at 2926 cm^−1^ and 1695 cm^−1^. The high intensity of these vibrations may be attributed to the assembly of amylose helices induced by starch treatment, which subsequently leads to the formation of RS. Similar absorption bands corresponding to C−C, C−OH, and C−H stretching vibrations were observed at 995 cm^−1^ and 1047 cm^−1^, indicative of ordered crystalline structures. The absorption peaks in the 1000 cm^−1^ region are associated with the vibrations of chemical bonds such as C−O−C and C−O−H, reflecting the backbone structure of starch molecules.

When comparing the FTIR spectra of RS from different lotus root germplasms, it was noted that the positions of the characteristic peaks were generally consistent, although differences in peak intensity were observed. For example, the intensity of the vibration peak at 2926 cm^−1^ was significantly lower in cooked RS compared to raw RS. This finding suggests that the C−H bonds were compromised during the preparation of cooked RS.

### 3.5. X-Ray Diffraction Pattern

**Figure 8:**
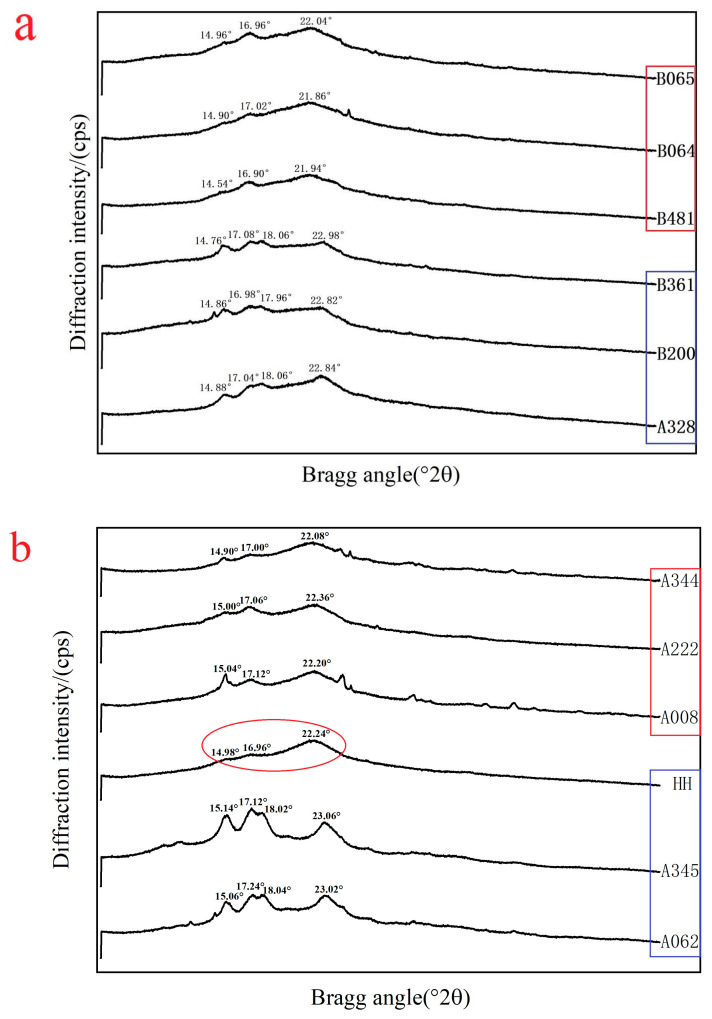
X-ray diffraction. Note: (**a**) Lotus seeds; (**b**) lotus rhizome. The samples within the red boxes are cooked resistant RS, and those within the blue boxes are raw RS.The purpose of the red circle in the figure is to highlight the differences.

X-ray diffraction (XRD) is a powerful technique for investigating the crystalline structure of lotus root RS. In the X-ray diffraction pattern (Figure 8), significant diffraction peaks are observed at 2θ angles of approximately 15°, 17°, 18°, 22°, and 23°. Among these, the peaks at 15°, 17°, 18°, and 23° correspond to the characteristics of the A-type crystalline structure, while the peaks at 17° and 22° align with the features of the B-type crystalline structure.

The diffraction pattern indicates that for raw RS, the peaks at four 2θ angles generally conform to the characteristics of the CA-type crystalline structure, except that the “HH” lotus root germplasm does not exhibit a distinct diffraction peak at 18°. In contrast, for cooked RS, all six lotus root germplasms show no distinct diffraction peak at 18°, which aligns more closely with the CB-type crystalline structure. Additionally, varying intensities of diffraction peaks are observed in all cooked RS patterns. This phenomenon may be attributed to high-temperature treatment during the preparation of cooked RS, which damages the original starch crystalline structure. Subsequently, amylose reassembles into double-helical structures and aggregates to form RS, resulting in lower crystallinity compared to the original raw RS structure.

The relative crystallinity values of RS from different lotus root germplasms, derived from their diffraction peak patterns, are presented in Table 3 and Table 4. Crystal size influences peak width; specifically, smaller crystal sizes result in wider peaks. A well-defined (sharp) peak indicates a surface with complete crystallization, suggesting that samples with high crystallinity exhibit sharper diffraction peaks. In the diffraction patterns, the peaks of raw RS are sharper than those of cooked RS. Raw RS, with its higher crystallinity, may exhibit greater resistance to digestive enzymes. This finding is significant for a deeper understanding of the digestive properties of lotus root RS and its applications in the food, pharmaceutical, and related fields.

## 4. Conclusions

The results showed that the RS content in raw lotus rhizome ranged from 1.03% to 20.40% (with an average of 13.86%) and significantly decreased to 0.63–8.07% (average: 1.89%) after cooking. For lotus seeds, the RS content in the raw state was 1.34–14.53% (average: 6.15%) and dropped to 1.08–4.90% (average: 2.20%) post-cooking. The data clearly indicated that the RS content in raw samples was significantly higher than that in cooked samples. Additionally, there were significant differences in RS content among different varieties (*p* < 0.05), demonstrating rich genetic diversity. Among the tested materials, germplasm A077, A265, A287, A345, B021, B189, B190, and B481 had significantly higher RS content than the population average. These high-RS materials provide valuable parental germplasm for breeding functional lotus varieties and exhibit excellent breeding potential.

The study found that the structures of RS from raw and cooked samples differed significantly. Raw RS had a compact surface structure, clear contours, and high crystallinity, with a crystal type of CA and sharp characteristic peaks. In contrast, after high-temperature treatment, cooked RS showed a loose structure, blurred boundaries, reduced crystallinity, and its crystal structure transformed into CB type with poor regularity. This structural change is mainly attributed to gelatinization and molecular chain rearrangement during cooking, which is consistent with the findings on banana RS in relevant literature.

A further comparison of RS between lotus seeds and lotus rhizome revealed differences in their particle morphology and crystal structure. Lotus seed RS had smaller particles and a rough surface, while lotus root RS had larger particles and a relatively smooth surface. FT-IR (Fourier Transform Infrared Spectroscopy) analysis showed slight differences in the intensity and shape of characteristic peaks between the two, reflecting variations in their molecular structures. XRD (X-ray diffraction) analysis confirmed that although both exhibited a typical C-type crystalline structure, the crystallinity of lotus seed RS was slightly higher than that of lotus root RS. These differences may be related to the distinct biological characteristics, growth locations, and starch synthesis of metabolic pathways of lotus seeds and lotus rhizome, which ultimately affect their anti-digestive properties and application potential in the food and nutrition fields.

## Figures and Tables

**Table 1:** Overall differences in RS content (%) in lotus germplasm (raw).

Category	Number of Germplasms	Minimum Value	Maximum Value	Range	Median	Mean	Standard Deviation	Coefficient of Variation
**Lotus Rhizomes**	95	1.03	20.40	19.37	12.58	13.86	3.93	28.35
**Lotus Seeds**	60	1.34	14.53	13.20	5.11	6.15	3.91	63.58

Note: The units of standard deviation and coefficient of variation are %.

**Table 2:** Overall differences in RS content (%) in lotus germplasm (ripe).

Category	Number of Germplasms	Minimum Value	Maximum Value	Range	Median	Mean	Standard Deviation	Coefficient of Variation
**Lotus Rhizomes**	95	0.63	8.07	7.43	1.53	1.89	1.33	70.40
**Lotus Seeds**	60	1.08	5.24	3.82	2.02	2.20	0.91	41.36

Note: The units of standard deviation and coefficient of variation are %. Correlation analysis.

**Table 3:** Relative crystallinity of lotus seed germplasm.

Cooked RS				Raw RS		
2θ	Relative Crystallinity			Relative Crystallinity		
	B064	B065	B481	A328	B200	B361
15°	1.921	2.838	1.084	1.039	1.811	2.188
17°	1.950	1.096	21.180	1.082	2.323	1.721
18°	/	/	/	38.364	1.758	0.986
22°	4.342	2.909	20.598	/	/	/

**Table 4:** Relative crystallinity of lotus root germplasm.

Cooked RS				Raw RS		
2θ	Relative Crystallinity			Relative Crystallinity		
	A008	A222	A344	A062	A345	HH
15°	3.804	1.850	2.693	2.868	3.935	2.600
17°	2.683	1.634	10.131	5.377	4.360	0.429
18°	/	/	/	2.953	3.660	/
22°	7.906	30.251	40.957	/	/	11.224

## Data Availability

The original contributions presented in this study are included in the article/Supplementary Materials. Further inquiries can be directed to the corresponding authors.

## Supplementary Materials

The following supporting information can be downloaded at https://www.mdpi.com/article/10.3390/polym18141737/s1. Table S1: Sampling Information Table of rhizome lotus samples Table S2: Sampling Information Table of lotus seeds samples Table S3: Quality determination results of starch in 95 lotus root germplasm (n = 3) Table S4: Determination results of starch quality of 60 lotus seed germplasm (n = 3).
