# Supplementary material for: Structure Identification of Germplasm Resources of Lotus with High Resistant Starch
Source: Polymers (Basel). 2026 Jul 15;18(14):1737. doi: 10.3390/polym18141737 (PMC13417616; doi:10.3390/polym18141737)
Supplement: Supplementary file 1 [file polymers-18-01737-s001.zip › polymers-4349907-supplementary.pdf]

Supplementary Materials

Table S1 Sampling Information Table of rhizome lotus samples

No.	Code	Sample Name	Harvest Time	No.	Code	Sample Name	Harvest Time
1	A002	Loyal Red Core	2023.04	49	A485	S93	2024.04
2	A008	Green Cloud	2023.04	50	A489	S95	2023.04
3	A034	Jiangxi Honglian	2023.04	51	A455	S78	2024.04
4	A036	Golden Light	2023.04	52	B003	B003	2024.04
5	A046	Beautiful Tricolor	2023.04	53	B005	B005	2024.04
6	A048	Nehru Lotus	2023.04	54	B015	B015	2024.04
7	A062	Pomegranate Red	2023.04	55	B019	B019	2024.04
8	A063	Dawn Light	2023.04	56	B021	B021	2024.04
9	A091	Caiyunfei	2023.04	57	B022	B022	2024.04
10	A121	Crane's Crest Red	2024.04	58	B029	B029	2024.04
11	A125	Red Lion	2023.04	59	B064	B064	2024.04
12	A143	Jinpan Chenglu	2023.04	60	B065	B065	2024.04
13	A151	Qianbanlian	2023.04	61	B104	B104	2024.04
14	A159	Sun Lotus	2023.04	62	B132	B132	2024.04
15	A165	Xiannu Sanhua	2023.04	63	B139	B139	2024.04
16	A169	Xueyuan Tingcui	2023.04	64	B140	B140	2024.04
17	A206	Red Silk Scarf	2023.04	65	B143	B143	2024.04
18	A209	Meizhong Red	2023.04	66	B144	B144	2024.04
19	A216	PT-B02	2023.04	67	B167	B167	2024.04
20	A217	PT-B03	2023.04	68	B168	B168	2024.04
21	A218	PT-B04	2023.04	69	B173	B173	2024.04
22	A219	PT-B05	2023.04	70	B175	B175	2024.04
23	A222	PT-B08	2023.04	71	B179	B179	2024.04
24	A223	PT-B09	2023.04	72	B185	B185	2024.04
25	A225	PT-B11	2023.04	73	B189	B189	2024.04
26	A244	PT-B30	2023.04	74	B195	B195	2024.04
27	A245	PT-B31	2023.04	75	B200	B200	2024.04
28	A248	PT-B34	2023.04	76	B233	B233	2024.04

Table S1(continued) Sampling Information Table of rhizome lotus samples

29	A249	PT-B35	2023.04	77	B245	B245	2024.04
30	A265	Radiant Belle	2023.04	78	B248	B248	2024.04
31	A287	Golden Monkey	2023.04	79	B267	B267	2024.04
32	A311	S06	2024.04	80	B271	B271	2024.04
33	A324	S12	2023.04	81	B275	B275	2024.04
34	A325	S13	2024.04	82	B277	B277	2024.04
35	A328	S14	2023.04	83	B278	B278	2024.04
36	A336	S18	2024.04	84	B279	B279	2024.04
37	A342	S21	2023.04	85	B280	B280	2024.04
38	A343	S22	2023.04	86	B281	B281	2024.04
39	A344	S22	2023.04	87	B320	B320	2024.04
40	A345	S23	2023.04	88	B350	B350	2024.04
41	A347	S24	2023.04	89	B353	B353	2024.04
42	A348	S24	2023.04	90	B362	B362	2024.04
43	A376	S38	2023.04	91	B447	B447	2024.04
44	A386	Dasajin	2023.04	92	B472	B472	2024.04
45	A405	S53	2023.04	93	B481	B481	2024.04
46	A420	S60	2024.04	94	HH	HH	2024.04
47	A458	S79	2024.04	95	XF	XF	2023.09
48	A462	S81	2023.04				

Table S2 Sampling Information Table of lotus seeds samples

No.	Code	Sample Name	Harvest Time	No.	Code	Sample Name	Harvest Time
1	A077	Z. Qingyun	2023.10	31	B173	B173	2023.10
2	A121	Hedinghong	2023.10	32	B175	B175	2023.10
3	A157	Sun Wen Lotus	2023.10	33	B179	B179	2023.10
4	A311	S06	2023.10	34	B185	B185	2023.10
5	A325	S13	2023.10	35	B189	B189	2023.10
6	A328	S14	2023.10	36	B190	B190	2023.10
7	A336	S18	2023.10	37	B193	B193	2023.10
8	A405	S53	2023.10	38	B195	B195	2023.10
9	A455	S78	2023.10	39	B200	B200	2023.10
10	A458	S79	2023.10	40	B233	B233	2023.10
11	A485	S93	2023.10	41	B245	B245	2023.10
12	B003	B003	2023.10	42	B247	B247	2023.10
13	B005	B005	2023.10	43	B248	B248	2023.10
14	B015	B015	2023.10	44	B267	B267	2023.10
15	B019	B019	2023.10	45	B271	B271	2023.10
16	B021	B021	2023.10	46	B275	B275	2023.10
17	B022	B022	2023.10	47	B277	B277	2023.10
18	B029	B029	2023.10	48	B278	B278	2023.10
19	B064	B064	2023.10	49	B279	B279	2023.10
20	B065	B065	2023.10	50	B280	B280	2023.10
21	B104	B104	2023.10	51	B281	B281	2023.10
22	B132	B132	2023.10	52	B320	B320	2023.10
23	B139	B139	2023.10	53	B335	B335	2023.10
24	B140	B140	2023.10	54	B350	B350	2023.10
25	B143	B143	2023.10	55	B353	B353	2023.10
26	B144	B144	2023.10	56	B361	B361	2023.10
27	B164	B164	2023.10	57	B362	B362	2023.10
28	B167	B167	2023.10	58	B447	B447	2023.10
29	B168	B168	2023.10	59	B472	B472	2023.10
30	B169	B169	2023.10	60	B481	B481	2023.10

Table S3 Quality determination results of starch in 95 lotus root germplasm（n=3）

Code	moisture（%）	raw RS（%）	cooked RS（%）	amylose（%）	amylopectin（%）
A002	67.37±0.03	17.23±0.08	1.21±0	20.54±0.01	79.46±0
A008	57.75±0.02	17.71±0.07	0.75±0.01	22.94±0.03	77.06±0.01
A034	62.39±0.04	17.23±0.05	0.88±0	21.6±0	78.4±0.01
A036	57.43±0	17.34±0.11	2.29±0.01	24.92±0	75.08±0.01
A046	57.59±0.01	18.25±0.03	0.77±0	28.76±0.01	71.24±0
A048	53.49±0.01	17.05±0.12	1.3±0.01	28.95±0	71.05±0
A062	59.13±0.02	17.74±0.05	0.79±0.01	30.1±0.02	69.9±0.01
A063	63.42±0	19.27±0.03	1.07±0.01	28.13±0	71.87±0
A091	51.71±0.01	16.05±0.01	1.59±0.02	32.1±0.02	67.9±0.02
A121	74.39±0	10.71±0.03	1.59±0.02	24.25±0.01	75.75±0.01
A125	57.4±0.01	18.24±0.06	0.94±0.01	36.31±0.01	63.69±0.02
A143	58.27±0.02	16.37±0.13	2.16±0.03	31±0	69±0
A151	56.64±0.01	15.34±0.15	1.07±0.01	30.64±0	69.36±0
A159	66.86±0.02	13.7±0.02	1.68±0.06	34.16±0.01	65.84±0
A165	54.46±0.02	18.61±0.07	2.04±0.02	31.37±0	68.63±0
A169	52.7±0.01	16.37±0.1	1.57±0.01	30.96±0.02	69.04±0
A206	57.78±0.01	14.82±0.05	1.34±0.03	33.45±0	66.55±0
A209	56.77±0	15.68±0.14	1.16±0.02	28.44±0	71.56±0
A216	54.94±0.01	16.31±0.08	1.86±0.03	32.95±0	67.05±0.01
A217	53.27±0.02	16.33±0.08	1.17±0.03	28.58±0	71.42±0
A218	58.79±0.01	16.33±0.17	2.26±0.02	28.69±0	71.31±0
A219	56.85±0	16.6±0.09	2.11±0.02	27.79±0.01	72.21±0
A222	61.59±0.01	16.47±0.03	4.6±0.01	36.99±0.01	63.01±0
A223	56.28±0	16.22±0.03	3.17±0.02	28.14±0.01	71.86±0
A225	52.35±0.01	18.23±0.05	1.13±0.02	28.8±0.02	71.2±0.01
A244	61.99±0.01	16.06±0.13	2.7±0.01	32.53±0.01	67.47±0.04
A245	53.77±0.01	16.25±0.05	3.94±0.03	30.26±0	69.74±0
A248	56.84±0.01	18.78±0.09	1.15±0.04	21.1±0.01	78.9±0
A249	55.12±0	19.11±0.06	2.64±0.01	28.62±0.01	71.38±0
A265	57.19±0.01	20.08±0.04	1.66±0.02	28.01±0.01	71.99±0
A287	85.36±0	19.43±0.01	1.26±0.01	24.93±0.01	75.07±0.03
A311	63.5±0.01	9.65±0.02	0.79±0.01	13.98±0.01	86.02±0.01
A324	84.8±0	18.89±0.11	1.08±0.03	28.2±0.01	71.8±0.01
A325	67.48±0	5.08±0.25	0.9±0	13.13±0	86.87±0.01
A328	71.81±0	19.31±0.05	1.33±0.03	30.86±0.02	69.14±0
A336	57.42±0	6.67±0.36	1.12±0.02	20.26±0.01	79.74±0.01
A342	62.88±0.01	18.59±0.05	0.9±0.01	30.8±0.01	69.2±0
A343	64.28±0	18.96±0.09	1.7±0.01	24.85±0.03	75.15±0.01
A344	64.22±0.02	18.81±0.02	1.48±0	19.44±0.02	80.56±0
A345	59.93±0.02	20.4±0.03	2.91±0.01	23.78±0.02	76.22±0

Table S3(continued) Quality determination results of starch in 95 lotus root germplasm（n=3）

Code	moisture（%）	raw RS（%）	cooked RS（%）	amylose（%）	amylopectin（%）
A347	60.58±0.01	18.41±0.12	1.89±0.01	22.82±0.01	77.18±0
A348	54.44±0.01	18.67±0.02	2.73±0.01	20.67±0.02	79.33±0.01
A376	53.33±0.01	18.34±0.02	1.73±0.03	28.67±0.01	71.33±0
A386	70.35±0.01	18.55±0.01	1.89±0.04	25.87±0	74.13±0
A405	66.4±0.01	10.39±0.01	1.4±0.02	16.37±0.01	83.63±0.02
A420	73.23±0	17.92±0.07	1.4±0.03	26.95±0.01	73.05±0.01
A455	77.15±0	9.52±0.14	0.63±0	17.82±0.01	82.18±0
A458	59.24±0.01	10.27±0.12	0.91±0	15.61±0.01	84.39±0
A462	73.81±0	18.48±0.03	3.11±0.03	21.89±0.01	78.11±0
A485	57.34±0.01	11.74±0.02	0.71±0	19.66±0.01	80.34±0
A489	77.54±0	18.77±0.04	1.16±0.01	25.79±0.01	74.21±0
XF	82.53±0	7.23±0.08	0.96±0.01	18.9±0	81.1±0
HH	70.59±0.01	2.77±0.02	1.42±0.01	21.02±0.02	78.98±0
B003	66.69±0	12.25±0.02	1.46±0.02	17.66±0.01	82.34±0
B005	73.45±0.02	11.08±0.02	0.91±0.01	16.52±0	83.48±0
B015	70.59±0	11.11±0.02	1.54±0.02	19.99±0	80.01±0.01
B019	79.73±0	11.42±0.03	3.54±0.01	15.71±0.03	84.29±0.02
B021	74.47±0	10.91±0.06	6.89±0.4	11.73±0	88.27±0.01
B022	78.04±0	11.84±0.02	5.56±0.1	14.44±0.01	85.56±0
B029	72.59±0.01	11.01±0.04	1.35±0.01	17.19±0	82.81±0.02
B064	75.7±0	9.62±0.07	1.63±0.02	14.08±0.03	85.92±0.01
B065	83.52±0	5.7±0.47	1.05±0.02	17.68±0.02	82.32±0.02
B104	67.14±0	10.67±0.06	1.52±0.02	14.69±0	85.31±0.01
B132	76.77±0	11.07±0.03	3.35±0.12	19.69±0	80.31±0
B139	69.15±0.01	11.05±0.01	3.89±0.08	13.27±0.01	86.73±0
B140	77.69±0	11.5±0	1.65±0.03	18.25±0.02	81.75±0.01
B143	80.93±0.01	11.43±0.02	1.26±0.04	13.72±0.01	86.28±0.01
B144	75.97±0	11.01±0.02	1.66±0.09	10.83±0.01	89.17±0.01
B167	78.59±0.07	10.52±0.05	0.97±0.01	13.34±0.01	86.66±0.01
B168	78.04±0.01	11.06±0.05	0.92±0.01	19.17±0.01	80.83±0
B173	81.55±0	8.9±0.01	0.72±0.01	13.37±0	86.63±0
B175	72.9±0	11.51±0.01	0.93±0.03	20.52±0.01	79.48±0.01
B179	61.74±0	12.58±0.06	1.91±0.05	29.14±0.02	70.86±0.01
B185	74.83±0.01	12.58±0.09	3.1±0.13	21.91±0.01	78.09±0.02
B189	66.3±0	13.66±0	8.07±0.04	22±0.01	78±0
B195	72.45±0	12.83±0.05	1.23±0.01	27.04±0.01	72.96±0.01
B200	71.41±0	12.45±0.02	1.33±0.01	23.71±0.01	76.29±0
B233	71.55±0	10.94±0.01	1.42±0.01	26.06±0	73.94±0
B245	69.8±0	11.14±0.05	1.65±0.01	21.02±0.03	78.98±0.02
B248	73.45±0	11.57±0	1.57±0.05	21.03±0	78.97±0
B267	71.87±0	11.45±0	3.14±0.08	20.76±0.01	79.24±0

Table S3(continued) Quality determination results of starch in 95 lotus root germplasm（n=3）

Code	moisture（%）	raw RS（%）	cooked RS（%）	amylose（%）	amylopectin（%）
B271	73.2±0	12.31±0	6.83±0.2	18.43±0.01	81.57±0
B275	73.25±0.01	11.2±0.01	2.35±0.06	17.23±0	82.77±0.01
B277	71.95±0	10.7±0.01	2.26±0.01	25.98±0.01	74.02±0.01
B278	87.84±0	11.12±0.01	2.1±0.04	17.99±0	82.01±0
B279	73.9±0	11.39±0.05	1.9±0.01	20.96±0	79.04±0
B280	71.37±0	10.99±0.02	1.4±0.01	19.64±0.01	80.36±0
B281	68.12±0	11.83±0	1.72±0.01	22.38±0.02	77.62±0.01
B320	72.39±0	11.74±0.02	2.15±0.02	27.35±0.02	72.65±0
B350	79.69±0.01	1.03±0	0.65±0.02	13.77±0	86.23±0
B353	73.75±0	11.39±0.01	1.58±0.01	17.69±0	82.31±0.01
B362	76.22±0	11.47±0.01	1.26±0.01	25.29±0	74.71±0.01
B447	78.6±0	11.14±0.05	1.79±0.01	20.15±0	79.85±0
B472	74.06±0	12.33±0.04	1.41±0.01	19.08±0	80.92±0.01
B481	68.97±0	12.71±0.02	1.26±0.01	22.9±0	77.1±0.01

Table S4 Determination results of starch quality of 60 lotus seed germplasm（n=3）

Code	moisture（%）	raw RS（%）	cooked RS（%）	amylose（%）	amylopectin(%）
A077	5.41±0.01	14.25±0.01	2.72±0	9.56±0.02	90.44±0.01
A121	5.97±0	12.43±0.34	1.32±0.02	9.29±0	90.71±0.02
A157	6.41±0	5.1±0.08	2.59±0	9.66±0.01	90.34±0
A311	4.41±0	12.16±0.02	1.84±0.01	9.62±0.01	90.38±0.01
A325	5.52±0.01	6.56±0.04	2.08±0.01	8.96±0.01	91.04±0.01
A328	4.22±0	14.53±0.03	3.45±0	8.31±0	91.69±0
A336	6.83±0	11.57±0.17	1.41±0.03	8.62±0.01	91.38±0.01
A405	2.98±0.01	10.73±0.25	1.7±0.01	7.82±0.01	92.18±0.01
A455	4.59±0.01	2.28±0.06	2.22±0.02	9.35±0.02	90.65±0.01
A458	3.45±0	8.16±0.09	2.84±0	9.35±0.01	90.65±0.01
A485	3.64±0	6.43±0.04	2.32±0.01	10.23±0.01	89.77±0.01
B003	6.08±0	11.13±0.05	2.45±0	9.28±0.01	90.72±0.01
B005	4.32±0	3.12±0.03	1.29±0	9.36±0.01	90.64±0
B015	4.92±0	8.9±0.06	1.72±0.01	8.49±0	91.51±0.01
B019	5.35±0	6.99±0.03	1.63±0.01	7.87±0.01	92.13±0.02
B021	5.24±0	9.26±0.09	1.66±0.02	7.72±0.01	92.28±0.01
B022	4.95±0	6.8±0.03	1.21±0.04	10.13±0.01	89.87±0.01
B029	5.28±0	8.69±0.08	3.11±0.01	9.91±0.01	90.09±0.01
B064	4.65±0	5.77±0.05	2.32±0.01	8.87±0.01	91.13±0
B065	5.2±0	11.67±0.13	1.08±0	9.7±0.01	90.3±0.01
B104	4.83±0	3.62±0.15	1.44±0.02	8.99±0	91.01±0.01
B132	5.7±0	5.12±0.14	1.61±0.01	12.06±0.01	87.94±0.01
B139	6.22±0	9.05±0.01	1.32±0.01	10.63±0.01	89.37±0.01
B140	6.52±0	3.71±0.1	2.63±0.01	9.55±0.01	90.45±0.01
B143	4.88±0	2.98±0.03	1.68±0	9.54±0.01	90.46±0.01
B144	5.67±0	2.11±0.01	1.67±0.02	8.37±0	91.63±0
B164	6.11±0	2.39±0.04	1.21±0	11.25±0	88.75±0.01
B167	5.17±0	3.1±0.12	1.7±0	10.95±0.01	89.05±0.01
B168	5.94±0	4.63±0.02	2.81±0	11.31±0.03	88.69±0.02
B169	4.89±0	1.52±0.01	3.85±0.03	10.03±0	89.97±0.01
B173	5.96±0	1.66±0.02	2.55±0.01	9.1±0.01	90.9±0.01
B175	5.89±0	3.23±0.1	4.29±0.01	8.53±0	91.47±0
B179	4.34±0	2.25±0.08	2.8±0.01	7.66±0.02	92.34±0
B185	5.72±0	4.07±0.03	2.21±0.02	8.72±0.02	91.28±0.02
B189	6.08±0	6.03±0.02	4.28±0.02	8.58±0.01	91.42±0
B190	5.96±0	11.14±0.27	4.9±0.01	9.45±0.02	90.55±0.01
B193	5.77±0	1.81±0.01	1.38±0	9.3±0.01	90.7±0
B195	5.93±0	1.79±0.02	2.43±0.01	8.43±0	91.57±0.01
B200	4.58±0	1.34±0	2.08±0.02	9.39±0.01	90.61±0.01
B233	5.42±0	4.83±0.12	2.64±0.01	7.85±0.02	92.15±0.01
B245	6.36±0	2.55±0.07	1.96±0.01	9.46±0.02	90.54±0.01

Table S4 (continued)Determination results of starch quality of 60 lotus seed germplasm（n=3）

Code	moisture（%）	raw RS（%）	cooked RS（%）	amylose（%）	amylopectin（%）
B247	6.63±0	2.49±0.07	1.72±0.01	9.22±0.01	90.78±0.01
B248	6.65±0	4.85±0.01	1.11±0.01	9.39±0	90.61±0.01
B267	5.23±0	2.47±0.01	1.7±0.02	10.04±0.01	89.96±0.01
B271	5.84±0	3.6±0	2.34±0.02	8.63±0.01	91.37±0.01
B275	6.64±0	7.46±0.02	1.39±0.03	9.76±0.01	90.24±0.01
B277	6.82±0	3.77±0.13	2.22±0.01	10.43±0.01	89.57±0.02
B278	6.78±0	7.61±0.03	2.35±0.01	9.91±0.01	90.09±0
B279	6.41±0	2.01±0.04	1.87±0.01	12.06±0.02	87.94±0.01
B280	6.31±0	3.19±0	2.53±0.01	10.8±0	89.2±0
B281	6.63±0	2.11±0.03	3.03±0.01	10.45±0.01	89.55±0
B320	6.18±0	3.16±0.01	2.81±0.01	11.15±0.01	88.85±0.01
B335	5.74±0	2±0.07	3.47±0.01	9.55±0.03	90.45±0.03
B350	5.73±0	2.67±0	2.91±0.01	9.1±0.01	90.9±0.01
B353	6.49±0	2.69±0	1.15±0.01	8.21±0.01	91.79±0
B361	5.65±0	4.48±0.12	2.96±0.01	8.98±0	91.02±0
B362	6.24±0	3.33±0.12	4.16±0.02	8.49±0.01	91.51±0
B447	6.61±0	3.88±0.01	4.41±0.01	8.64±0.01	91.36±0.01
B472	6.73±0	5.22±0.08	4.4±0	8.96±0.01	91.04±0
B481	6.53±0	8.56±0.08	5.24±0.02	7.96±0.01	92.04±0.01
